# 12.4 BRAIN LACTATE IS RELATED TO COGNITION IN SCHIZOPHRENIA

**DOI:** 10.1093/schbul/sby014.047

**Published:** 2018-04-01

**Authors:** Laura Rowland, Andrea Wijtenburg, Subechhya Pradhan, Stephanie Korenic, Richard Edden, Elliot Hong, Peter Barker

**Affiliations:** 1 University of Maryland School of Medicine; 2 Maryland Psychiatric Research Center; 3 Johns Hopkins University

## Abstract

Laura Rowland, University of Maryland School of Med: Bioenergetic function may be altered in schizophrenia as supported by post-mortem, preclinical, cerebrospinal fluid, and 31P-magnetic resonance spectroscopy (MRS) research. Impairments in bioenergetic function may lead to cognitive and functional dysfunction, characteristics of the illness. First, a 7T MRS study that tested the hypothesis that frontal lactate concentrations are elevated in schizophrenia and related to cognitive impairments will be presented. Second, recent advances in brain lactate measurements with 3T MRS will be presented.

**Methods:**

Twenty-nine controls and 27 participants with schizophrenia completed the study. MRS scanning was conducted on a Philips ‘Achieva’ 7T scanner, and spectra were acquired from a frontal voxel using STEAM (TE/TM/TR=14/33/3000 ms, 128 NEX, 16 NEX water). Participants completed the MATRICS Consensus Cognitive Battery (MCCB) for cognitive function and UCSD Performance-Based Skills Assessment (UPSA) for functional capacity. The relationships between lactate, MCCB, and UPSA were examined. 3T MRS test-retest measures of lactate were conducted on a Siemens Prisma scanner using spectra editing (TE/TR=140/3, editing pulse at 4.1ppm with 30Hz bandwidth, 360 NEX, 16 NEX water).

**Results:**

Patients had significantly higher lactate compared to controls (p = 0.045). Higher lactate was associated with poorer general cognitive function (r=-0.36, p=0.01) Visual learning, processing speed, and reasoning/problem solving cognitive domains showed the strongest relationships with lactate. Poorer functional capacity (r=-0.43, p=0.001) was also related to higher lactate. 3T spectral editing studies showed excellent reproducibility with a mean coefficient of variation of 4%.

**Discussion:**

Higher frontal lactate levels in schizophrenia support the hypothesis that brain bioenergetics are altered and related to cognitive and functional impairments in schizophrenia. Higher lactate could be due to inefficient aerobic metabolism causing a shift towards anaerobic metabolism or poor utilization of lactate. Lactate measurements are doable at 3T field strength and may be a useful biomarker of cognition in schizophrenia. Interventions to promote efficient mitochondrial energy metabolism may prove useful for enhancing cognition and alleviating functional impairments in schizophrenia.

